# Effect of urinary sodium-to-potassium ratio change on blood pressure in participants of the longitudinal health of adults study - ELSA-Brasil

**DOI:** 10.1097/MD.0000000000016278

**Published:** 2019-07-12

**Authors:** Taísa Sabrina Silva Pereira, José Geraldo Mill, Rosane Harter Griep, Rosely Sichieri, Maria del Carmen Bisi Molina

**Affiliations:** a Department of Health Sciences, University of the Americas Puebla, Puebla, México; b Post Graduate Programme in Public Health, Federal University of Espírito Santo, Espírito Santo; c Laboratory of Health and Environment Education, Oswaldo Cruz Fundation; d Institute of Social Medicine, State University of Rio de Janeiro, Rio de Janeiro, Brazil.

**Keywords:** blood pressure, potassium, sodium, sodium-to-potassium ratio

## Abstract

To assess the effect of changing the sodium to potassium (Na/K) ratio on blood pressure at 4 years of follow-up.

The measurements were carried out under identical conditions in two study periods (2008–2010 and 2012–2014). Urinary excretion of sodium and potassium (mmol/L) over 12 nocturnal hours was used to calculate the Na/K ratio and categorized by quintile. The 24-hour sodium and potassium intake was estimated using a validated equation. The mean BP was calculated from 3 measurements after 5 minutes of rest. Of the 15,105 participants at baseline, 14,014 completed the first follow-up. Participants without validated urine collection (n = 5,041), using antihypertensive medication (n = 3,860) at either time points or reporting bariatric surgery during follow-up (n = 45) were excluded. The differences between follow-up and baseline values were calculated for BP and the Na/K ratio. Analyses were stratified by sex and adjusted for confounding variables.

Sodium intake did not change from baseline, but potassium intake increased by approximately 150 mg in both sexes (*P* < .001), with a consequent reduction of the Na/K ratio. The highest quintile of change in the Na/K ratio was associated with greater variation in BP. When adjusted for covariates, it is possible to observe an increase in SBP in women from the third quintile of the Na/K ratio, in men this increase was observed from the fourth quintile. However, for DBP this increase is observed from the third quintile in both men and women.

Increase in SBP was observed in women from the third quintile of the Na/K ratio, in men this increase is observed from the fourth quintile. However, for DBP this increase is observed from the third quintile in both men and women. The Na/K ratio demonstrated a greater association in BP.

## 1 Introduction

Arterial hypertension (AH) is an independent risk factor for cardiovascular diseases, particularly for coronary artery disease and stroke,^[1,2]^ which are leading causes of mortality worldwide.^[3]^ Several factors may influence AH development, including genetic^[4]^ and lifestyle^[5]^ factors. Of particular interest among these risk factors, are diets with high sodium and low potassium intake.^[6]^

Sodium and potassium have independent effects on blood pressure (BP). Excessive sodium intake leads to fluid retention, increased extracellular volume and elevated BP.^[7]^ In contrast, higher plasma levels of potassium stabilize the membrane potential in vascular smooth muscle cells, reducing the peripheral vascular tone and resistance and consequent decrease in BP.^[8]^ Thus, diets low in sodium and high in potassium act synergistically to lower BP and the prevalence of AH. For this reason, the sodium/potassium (Na/K) ratio in urine has been used as an indicator of diet quality in relation to BP control.^[9]^ In addition, a higher Na/K ratio has also been used as an indication of higher intake of processed foods and higher salt addition in food preparation.^[9]^ Oliveira et al^[10]^ show that the frequent intake of industrialized condiments is associated with higher salt excretion and Na/K ratio in Brazilian individuals, as well as higher ultraprocessed consumption increases Na/K ratio. Higher Na/K ratios are also indicative of lower intake of fruits and vegetables, which are important sources of potassium.^[9]^

The World Health Organization (WHO) recommends that the Na/K ratio be approximately 1, which can be achieved by reducing salt intake and by maintaining adequate daily intake of fresh fruits and vegetables.^[11]^ Decreasing sodium intake by reducing intake of processed and ultraprocessed foods can also help to reach the goal recommended by the WHO.^[12]^ In a study in Vitória / ES, Brazil, the urinary Na/K ratio was 5.1 ± 3.6 for men and 4.8 ± 2.8 for women.^[13]^ In the Brazilian Longitudinal Study of Adult Health (ELSA-Brasil) population, men showed higher mean values (4.1 ± 2.0) of the Na/K ratio compared to women (3.6 ± 1.7).^[14]^ All these values well above the WHO recommendation for this variable, which is approximately 1.^[11]^

Several studies have demonstrated a positive association between urinary Na/K ratio and BP and prevalence of cardiovascular diseases. A cohort study in Chinese showed that the Na/K ratio was more strongly associated with the incidence of AH than either sodium or potassium alone.^[15]^ A meta-analysis showed that increases in potassium and lower Na/K ratios were associated with lower BP^[16]^; however, when combining studies from different countries, Mente et al^[17]^ found a non-linear association between the Na/K ratio and BP, with a more pronounced association among people with a high level of sodium intake. Sodium intake in Brazil (3.1 g/day) even when stratified by sex (men = 3.5 g/day; women = 2.8 g/day)^[18]^ exceeds the recommendation proposed by the WHO that is 2.3 g/day equivalent to 5 g of salt/day.^[11]^ In the ELSA-Brasil population, the sodium intake was 4.2 g /day.^[19]^

The present study considers that the increase of this relationship occurs both by the increase of the consumption of sodium and of industrialized products, as well as by a decrease in the consumption of potassium present in fruits and vegetables. Our purpose was to assess the effect of changing the sodium to potassium (Na/K) ratio on blood pressure at four years of follow-up.

## 2 Methods

### 2.1 Population and study design

Data were obtained from the ELSA-Brasil, a longitudinal study in a cohort of 15,105 active or retired public servants of both sexes, aged 35 to 74 years old, from 5 higher education institutions and one research institution.^[20]^ The study was approved by the Research Ethics Committees of each institution involved (Federal University of Rio Grande do Sul; University of São Paulo, Oswaldo Cruz Foundation, Federal University of Minas Gerais, Federal University of Espírito Santo and Federal University of Bahia), and all participants signed an informed consent form. At the scheduled date, participants attended one of the 6 research centres for clinical and laboratory tests and completed questionnaires via an interview.^[20]^

For this study, the data collected at baseline (2008–2010) and at the first follow-up (2012–2014) were analyzed, with a mean interval of 3.8 years. All the protocols used in both study periods followed the same criteria. Since antihypertensives and diuretics mask the real BP values, the main outcome of this study, participants who reported using these drugs at baseline or follow-up were excluded from this analysis. Participants who had undergone bariatric surgery during this time were also excluded, because in these patients usually produces an important BP decrease, mainly in presence of AH.

### 2.2 Data collection

A 12-hour nocturnal urine collection was performed at baseline and follow-up to estimate renal function and excretion of sodium and potassium. Overnight 12-hour urine collection was previously validated to estimate the daily sodium and potassium intake.^[21]^ The 12-hour urinary may be used in epidemiological studies because it reliably estimates the habitual consumption of sodium and potassium since good agreement was observed among the 5 measurements evaluated over a one year (sodium ICC 0.65, *P* < .001; potassium ICC 0.54, *P* < .001).^[22]^

Participants received verbal and written information regarding the collection of urine, as well as a 2-litre plastic bottle when the exam was scheduled. They were asked to perform the urine collection between 7:00 pm and 7:00 am the next morning and to note the exact time of beginning and end of collection as well as any urine loss. The records were received by the team of researchers along with the collected urine on the day of the exams. Urine aliquots were sent to the Central Laboratory of ELSA-Brasil for creatinine estimation by the Jaffé method and measurement of electrolytes by the selective ion electrode method – ISE^[23]^ (1 mmol/L precision).

### 2.3 Exposure

The 12-hour urine collection was considered valid if it simultaneously met three criteria: I) collection time between 10 and 14 hours, II) volume collected equal to or greater than 250 mL, and III) creatinine excretion, adjusted for 12 hours and corrected by body weight, between 7.2 and 16.8 mg/kg in men and between 5.4 and 12.6 mg/kg in women.^[24]^ The Na/K ratio was calculated using sodium and potassium (mmol/L) concentrations over 12 hours. Daily intake of sodium and potassium was also estimated from the 12-hour urinary excretion according to a previously validated method.^[21]^ The change in Na/K ratio was estimated as the difference (Δ) between the Na/K measured at follow-up and that measured at baseline. Values for the change in Na/K ratio were categorized into quintiles, where the lowest Δ was considered the 1st quintile and the highest Δ was considered the 5th quintile.

### 2.4 Outcome

BP was measured under strict standardized procedures using an Omron automatic device (HEM 705CPINT)^[25]^ after at least 5 minutes of rest in a temperature-controlled room (20–24°C). Participants had to have an empty bladder, be sitting upright with their back relaxed and supported on the chair, without crossing their legs and with their feet resting on the floor, and their left arm in the mobile armrest and free of clothing. For each participant, 3 measurements were obtained in the left arm with an interval of 2 minutes between measurements. The arithmetic mean of the last two measurements was used for the analysis.^[20,25]^ BP was considered elevated for systolic BP (SBP) values ≥140 mmHg and diastolic BP (DBP) values ≥90 mmHg. The change in SBP and DBP was calculated as the Δ between SBP and DBP measured at follow-up and that measured at baseline.

### 2.5 Covariates

The independent variables used in this study were age (years), highest level of schooling (elementary incomplete, elementary, secondary school and higher education). Race/skin colour (black, brown, white and other–Asian or indigenous) was self-reported. Per capita income was calculated from the approximate net family income during the month prior to the interview and the number of people who depended on this income.

The body mass index (BMI) was calculated based on the ratio between weight and height. An electronic scale (Toledo, model 2096PP) with a capacity of 200 kg and a precision of 50 g was used to measure body weight. Height was measured using a wall stadiometer (Seca, Hamburg, BRD) with a precision of 0.1 cm.^[25]^

Smoking history was categorized as never smoker, former smoker and current smoker. The alcohol intake was estimated by using a questionnaire and given as grams of ethanol per week.^[26]^ Physical activity was estimated from the International Physical Activity Questionnaire (IPAQ). This consists of questions relating to the frequency and duration of physical activities (walking and moderate or vigorous exercise) that are developed at work, in going from place to place (commuting), in domestic activities and during leisure time.^[27]^ In ELSA-Brasil, only the domains of leisure time and commuting were evaluated. The physical activity pattern was reported in minutes/week, and only physical activity that was performed for at least 10 minutes/week was considered.

Differences (Δ) in continuous variables (age, income, alcohol intake, physical activity, and BMI) were calculated as the difference between the measurement obtained at follow-up and that obtained at baseline.

### 2.6 Data analysis

Analyses were stratified by sex because the intake of these nutrients is different in women and men. Means and standard deviations were calculated for sodium, potassium, Na/K ratio, SBP and DBP at the 2 study visits and comparisons were made using the paired *t* test. Univariate covariance analysis (ANCOVA) was used to evaluate the effect of age on change in SBP and DBP. Crude and adjusted multivariate linear regression models were constructed, adjusting for qualitative baseline characteristics (race/skin color), follow-up variables (age, schooling, smoking), and Δ income, alcohol intake, physical activity and BMI. We adjusted for covariates in two models as follows: Model 1: race/skin colour, age, schooling, income per capita; Model 2: Model 1 + smoking, alcohol intake, physical activity and body mass index. Data were analyzed using the statistical software Statistical Package for Social Sciences- SPSS 23.0, and the significance level adopted for all tests was α ≤ 5%.

## 3 Results

**Figure 1:**
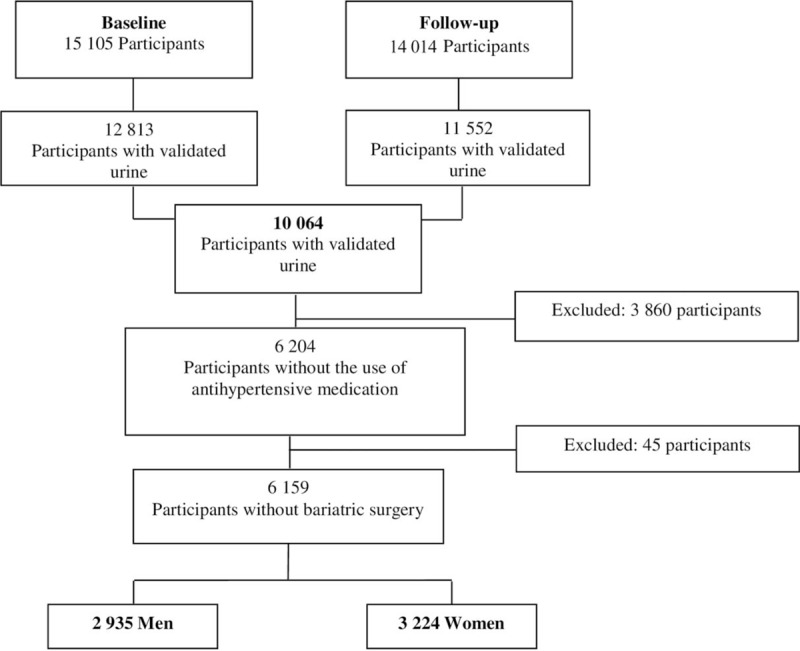
Exclusion Flow Chart for Elsa-Brasil participants.

Of the 15,105 participants included at baseline, 14,014 completed the follow-up evaluation. Reasons for loss to follow-up (5.9%) included as loss of contact or refusal to participate (n = 887) and death (n = 204, 1.3%). A total of 10,064 participants showed valid 12-hour urine collections at both time points. Of these, 3860 participants were using antihypertensive medication and 45 individuals reported having undergone bariatric surgery during the follow-up interval. Thus, the final sample consisted of 6.159 participants, of whom 2935 (47.6%) were men and 3,224 (52.4%) were women (Fig. 1).

The distribution of self-reported race/color was 55.9% white, 12.2% black, 27.2% brown and 3.6% other. Among those who reported having modified their diet in the preceding 6 months at follow-up, 23.9% (n = 701) were men and 32.5% (n = 1049) were women.

From baseline to follow-up income, alcohol intake, BMI and the proportion of elevated BP increased in both sexes. However, the proportion of smokers decreased among both men and women. Physical activity decreased among men, whereas it increased among women (Table 1).

**Table 1:**
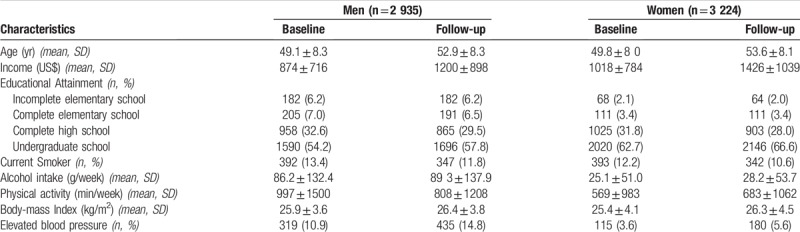
Sociodemographic, lifestyle and health characteristics of Baseline participants (2008–2010) and Follow-up (2012–2014) of ELSA-Brasil.

Estimated potassium intake increased (*P* < .001) among both men and women from baseline to follow-up decreasing the Na/K ratio whereas both SBP and DBP increased (*P* < .001) (Table 2).

**Table 2:**
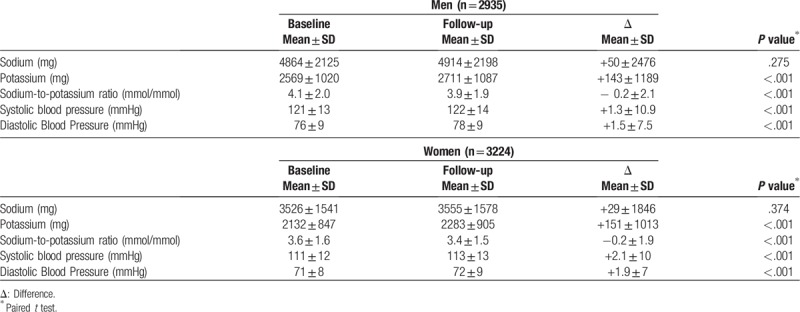
Means, standard deviation (SD) and difference between intake measures and hemodynamic variables among the participants of ELSA-Brasil (2008–2010 and 2012–2014).

**Figure 2:**
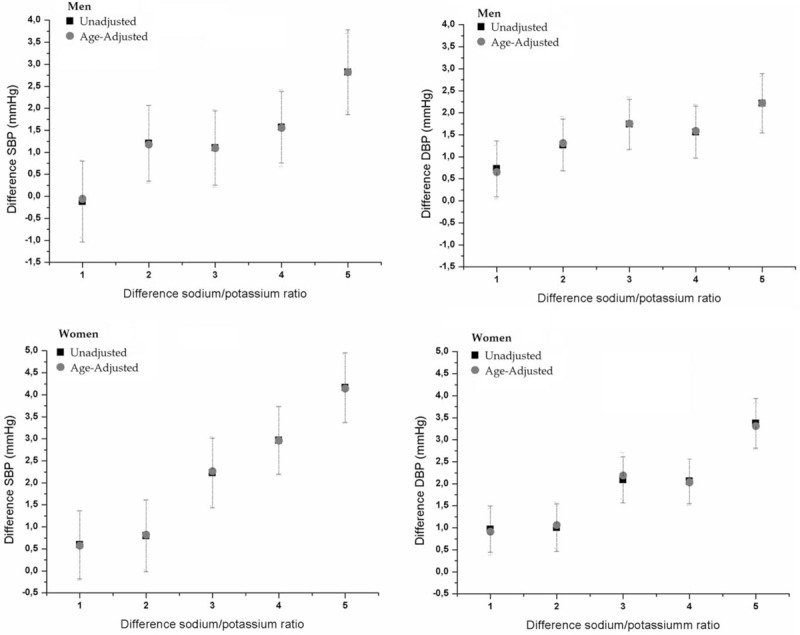
Differences (unadjusted and age-adjusted) of systolic blood pressure (SBP) and diastolic (DBP) between of quintiles of variation of sodium-to-potassium ratio in men and women participating in ELSA-Brasil (2008–2010 and 2012–2014).

Figure 2 shows the change in BP as a function of the quintiles of change in Na/K ratio. In both men and women, an increase in the Na/K ratio (higher quintiles) was associated with a higher increase in SBP than DBP. The effect on BP was more pronounced in men. When adjusted for age using the ANCOVA test, we observed that it maintained the same tendency to increase BP according to the quintiles of the Na/K ratio.

In general there is a more than linear increase of SBP and DBP with of Na/K ratio. Variations for the second quintile compared to first were less pronounced (Table 3). The β coefficient for the Δ in SBP increased with each quintile of Δ in Na/K ratio, both in the crude model and in the adjusted models. For Δ in DBP, a dose-response effect was observed only in the crude model. After adjustment, participants in the 5th quintile still had the largest Δ in DBP (Table 3). When adjusted for covariates, it is possible to observe an increase in SBP in women from the third quintile of the Na/K ratio, in men this increase was observed from the fourth quintile. However, for DBP this increase is observed from the third quintile in both men and women.

**Table 3:**
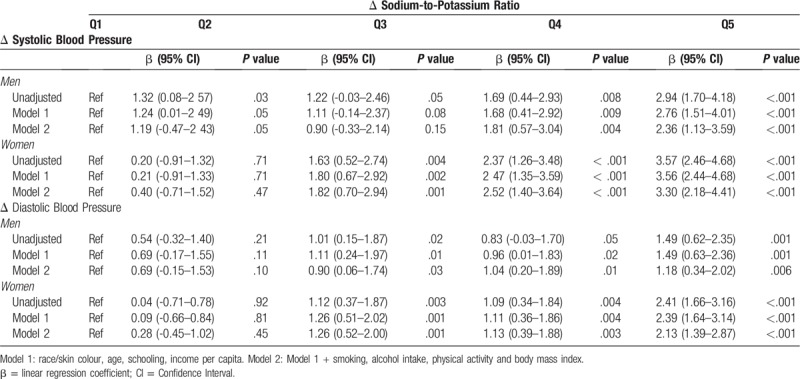
Regression coefficient of four year blood pressure change unadjusted and adjusted∗ by quintiles of urinary sodium-to-potassium ratio and sex among the participants of ELSA-Brasil (2008–2010 and 2012–2014).

## 4 Discussion

In this study, we observed a linear association between urinary Na/K ratio and BP increase over a mean follow-up of 3.8 years in both men and women, being that the increase in BP was observed from the third quintile of the Na/K ratio adjusted by race/skin color, age, schooling, income per capita, smoking, alcohol intake, physical activity and body mass index. We found independent associations of sodium and potassium intake with BP, but when evaluated in a combined manner, as in the case of the Na/K ratio, the effect was potentiated. The superiority of the Na/K ratio as a predictor of BP variation compared to sodium and potassium as individual predictors has been observed in several studies, most of them conducted in hypertensive patients.^[28]^ Our work confirms this finding in a sample composed predominantly by a large number of normotensive individuals.

Excess sodium has been associated with increased BP levels,^[29,30]^ arterial stiffening^[30]^ and with the risk of developing hypertension.^[31]^ It is important to consider that the increase in BP levels due to high salt intake varies across individuals. Molina et al.^[13]^ in a cross-sectional study, demonstrated that salt intake and Na/K ratio were positively associated with increased BP levels.

The individual BP response to salt intake depends on sodium sensitivity, with some individuals experiencing a considerable increase in BP, whereas others do not. Diabetics, those with chronic kidney disease, the elderly and Blacks tend to be more sensitive to salt.^[32]^ These patterns are likely even more complex in the Brazilian population, due to its ethnic diversity, as African genetic descent contributes to a greater blood pressure increase with age.^[33]^ Consequently, the increase in the risk of developing AH at some point in life is greater in black individuals. Therefore, there is sufficient evidence supporting the need for sodium intake reduction in order to reduce cardiovascular risk in populations with high salt intake.

Bernstein and Willett^[34]^ evaluated sodium intake in the American population from 1957 to 2003, concluding that there were no changes in intake and that intake levels remained above recommended levels. In Brazil, there are no estimates of intake over time, but the same trend was observed in this study. In the first period of the study, carried out in 2008 to 2010, the estimated salt intake was 10.5 g/day,^[19]^ and after approximately 4 years, it was 10.8 g/day. However, potassium intake increased by approximately 147 mg/day between the two study periods.

A meta-analysis of 33 studies conducted in adults and 2 studies in children found that high potassium intake reduced SBP by 5.93 mmHg and DBP by 3.78 mmHg.^[35]^ A systematic review and meta-analysis of clinical trials of potassium supplementation in hypertensive patients found that high potassium intake decreased BP.^[36]^ Another meta-analysis performed with data from 14 cohorts showed an inverse and significant association between potassium intake and the risk of stroke. Another study found that for each 1-g increase in daily potassium intake, there was a reduction of 10% in the risk of stroke.^[37]^

We found no significant variation with potassium alone, possibly due to the low intake of fruits and vegetables in this population. Urinary excretion of potassium was positively correlated with quality of diet,^[38]^ since the main sources of potassium are fruits and vegetables.^[8]^ A meta-analysis with prospective studies by Wu et al^[39]^ demonstrated a reduction in the risk of hypertension with greater intake of fruits and vegetables. Other studies also found inverse associations of fruit and vegetable intake with hypertension,^[40–42]^ stroke^[43]^ and coronary diseases.^[44]^

A review study by Iwahori et al^[9]^ presents the Na/K ratio as a summary index to evaluate sodium reduction and potassium increase in dietary changes. The lower Na/K ratio indicates - low sodium intake and high potassium intake, characterizing a diet of better nutritional quality. When the sociodemographic factors with the highest Na/K ratio we evaluated, it was observed that men, young people and individuals with lower schooling and income, being that these characteristics showed higher Na/K ratio.^[14]^ In the present study we observed that the highest Na/K ratio was presented in men, corroborating with other studies.^[13,14]^

Diets with high Na/K ratio are directly associated with the higher incidence of AH, as observed by Du et al^[15]^ in a Chinese adult cohort. In Australia, the Na/K ratio was positively associated with SBP in individuals aged 50 to 75 years. Participants were also classified according to the quintile of the Na/K ratio, and those in the highest quintile had a 52% prevalence of AH, while those in the first quintile had a prevalence of 36%.^[29]^ Study conducted with participants of the *Korea National Health and Nutrition Examination Survey (*KNHANES), adults aged 20 to 79 years, evaluated the Na/K ratio with hypertension. The authors observed that the last quartile of the Na/K ratio. presented the highest prevalence of AH.^[45]^

In Brazil, a population-based study conducted in Vitória / ES, evaluated 12-hour urine excretion and observed the highest Na/K ratio was associated with higher SBP and DBP when compared to a lower Na/K ratio.^[46]^

Sensitivity analysis showed that participants who were excluded from the present analysis (those taking anti-hypertensive medication and previous bariatric surgery) did not differ in relation to sex distribution. However, they differ from those included in our analyses in terms of schooling, smoking, alcohol consumption and physical activity practice (lower in the excluded group). Conversely, they showed higher income, age and BMI. Individuals on antihypertensive medication were older 3.8 ± 0.6 years) and showed higher SBP and DBP, as well as higher sodium, potassium and Na/K ratio than those without medication. Despite the difference in average age, subjects using the medication and higher Na/K ratio, it shows that despite using medication, the behavior in relation to the diet is different. The regression result follows the same pattern as presented in the present study, the last quintile of the relationship being the one that most increases SBP and DBP.

The relevance of this study is the longitudinal analysis of a population in a middle-income country with high sodium intake and low potassium intake. Another important strength is the method used to estimate the Na/K ratio, which is considered the gold standard.^[47–49]^ Therefore, the Na/K ratio can be used as an indicator of the consumption of these electrolytes, since they do not present biases as found in food methods. However, this indicator is not able to identify the food consumed by the population, what can be considered a limitation on this method. Thus, even though the urinary Na/K ratio does not allow to identify the food sources, nor what contributes to elevate it, we can affirm that the higher this ratio, the more frequent is the consumption of processed condiments and processed foods.^[10]^ Although urinary excretion is a point marker of these electrolyte,^[47]^ a study conducted by our research group shows that urinary 12-hour sodium and potassium excretion can be considered as an estimate of habitual intake for the population, since there was no variation in measurements performed over a year-long period.^[22]^ Errors of urine collection, if occurred, would tend to bias any observed association between Na/K ratio with SBP or DBP toward the null rather than create a spurious relationship.

Although urine collection is an uncommon procedure in clinical practice, ELSA-Brasil maintains high methodological rigor and quality control of its measurements, with standard training in all the research centers for the orientation of participants, evaluation of clinical tests and administration of questionnaires.^[50]^

In conclusion, the Na/K ratio is positively associated with increased BP, an increase in SBP was observed in women from the third quintile of the Na/K ratio, in men this increase is observed from the fourth quintile. However, for DBP this increase is observed from the third quintile in both men and women. The Na/K ratio demonstrated a greater association in BP, which wasn’t observed when the effects of these electrolytes were analyzed separately.

## Acknowledgments

The authors thank the staff and participants of the ELSA-Brasil Study for their important contributions.

## Author contributions

**Conceptualization:** Taísa Sabrina Silva Pereira, José Geraldo Mill, Maria del Carmen Bisi Molina.

**Data curation:** Taísa Sabrina Silva Pereira.

**Formal analysis:** Taísa Sabrina Silva Pereira.

**Methodology:** Taísa Sabrina Silva Pereira, Maria del Carmen Bisi Molina.

**Writing – original draft:** Taísa Sabrina Silva Pereira.

**Writing – review & editing:** Taísa Sabrina Silva Pereira, José Geraldo Mill, Rosane Harter Griep, Rosely Sichieri, Maria del Carmen Bisi Molina.

## References

[1] Global Burden of Metabolic Risk Factors for Chronic Diseases Collaboration. Cardiovascular disease, chronic kidney disease, and diabetes mortality burden of cardiometabolic risk factors from 1980 to 2010: a comparative risk assessment. Lancet Diabetes Endocrinol 2014;2:634–47. 24842598 10.1016/S2213-8587(14)70102-0 PMC4572741

[2] Malachias MVB Plavnik FL Machado CA 7a Diretriz Brasileira de Hipertensão Arterial: Capítulo 1 - Conceituação, epidemiologia e prevenção primária. Arq Bras Cardiol 2016;107:1–6. 10.5935/abc.20160151 PMC5319472 27819380

[3] World Health Organization. Global status report on noncommunicable diseases 2014. Geneva, Switzerland: World Health Organization; 2014.

[4] Windham BG Griswold ME Lirette S Effects of age and functional status on the relationship of systolic blood pressure with mortality in mid and late life: the ARIC study. J Gerontol A Biol Sci Med Sci 2015;72:89–94. 26409066 10.1093/gerona/glv162 PMC5155654

[5] Geleijnse JM Kok FJ Grobbee DE Impact of dietary and lifestyle factors on the prevalence of hypertension in Western populations. Eur J Public Health 2004;14:235–9. 15369026 10.1093/eurpub/14.3.235

[6] Zhang Z Cogswell ME Gillespie C Association between Usual Sodium and Potassium Intake and Blood Pressure and Hypertension among U.S. Adults: NHANES 2005–2010. Plos One 2013;8:e75289. 24130700 10.1371/journal.pone.0075289 PMC3794974

[7] Morrison AC Ness RB Sodium intake and cardiovascular disease. Annu Rev Public Health 2011;32:71–90. 21219163 10.1146/annurev-publhealth-031210-101209

[8] Weaver CM Potassium and Health. Adv Nutr 2013;4:368S–77S. 23674806 10.3945/an.112.003533 PMC3650509

[9] Iwahori T Miura K Ueshima H Time to consider use of the sodium-to-potassium ratio for practical sodium reduction and potassium increase. Nutrients 2017;9:E700. 28678188 10.3390/nu9070700 PMC5537815

[10] Oliveira LS Coelho JS Siqueira JH Relación sodio/potasio urinario y consumo de condimentos industrializados y alimentos ultraprocesados. Nutr Hosp 2019;36:125–32. 30834771 10.20960/nh.02101

[11] World Health Organization. Diet, Nutrition and the Prevention of Report of a Joint WHO /FAO Expert Consultation. Geneva, Switzerland 2003.

[12] Brasil. Ministério da Saúde. Secretaria de Atenção à Saúde. Departamento de Atenção Básica. Guia Alimentar para a População Brasileira. 2° ed. Brasília. 2014.

[13] Molina MCB de Sá Cunha R Herkenhoff LF Hipertensão arterial e consumo de sal em população urbana. Rev Saude Publica 2003;37:743–50. 14666304 10.1590/s0034-89102003000600009

[14] Pereira TSS Mill JG Cade NV Griep RH Sichieri R Molina MCB Fatores associados à relação sódio/potássio urinária em participantes do ELSA-Brasil. Cad Saude Publica “in press”. 10.1590/0102-311X00039718 31340331

[15] Du S Batis C Wang H Understanding the patterns and trends of sodium intake, potassium intake, and sodium to potassium ratio and their effect on hypertension in China. Am J Clin Nutr 2014;99:334–43. 24257724 10.3945/ajcn.113.059121 PMC3893725

[16] Binia A Jaeger J Hu Y Daily potassium intake and sodium-to-potassium ratio in the reduction of blood pressure: a meta-analysis of randomized controlled trials. J Hypertens 2015;33:1509–20. 26039623 10.1097/HJH.0000000000000611

[17] Mente A O’Donnell MJ Rangarajan S Association of urinary sodium and potassium excretion with blood pressure. N Engl J Med 2014;371:601–11. 25119606 10.1056/NEJMoa1311989

[18] de Moura Souza A Bezerra IN Pereira RA Dietary Sources of Sodium Intake in Brazil in 2008–2009. J Acad Nutr Diet 2013;113:1359–65. 23830323 10.1016/j.jand.2013.04.023

[19] Pereira TSS Benseñor IJM Meléndez JGV Sodium and potassium intake estimated using two methods in the Brazilian Longitudinal Study of Adult Health (ELSA-Brasil). São Paulo Med J 2015;133:510–6. 26760125 10.1590/1516-3180.2015.01233108 PMC10496553

[20] Aquino EML Barreto SM Bensenor IM Brazilian Longitudinal Study of Adult health (ELSA-Brasil): objectives and design. Am J Epidemiol 2012;175:315–24. 22234482 10.1093/aje/kwr294

[21] Mill JG da Silva ABT Correlation between sodium and potassium excretion in 24- and 12-h urine samples. Braz J Med Biol Res 2012;45:799–805. 22782553 10.1590/S0100-879X2012007500114 PMC3854318

[22] Molina MCB Pereira TSS Porto AS Validation of the single measure of 12-hour urine excretion for estimation of sodium and potassium intake. Sao Paulo Med J 2018;136:150–6. 29694492 10.1590/1516-3180.2017.0210031117 PMC9879545

[23] Fedeli LG Vidigal PG Leite CM Logística de coleta e transporte de material biológico e organização do laboratório central no ELSA-Brasil. Rev Saúde Pública 2013;47:63–71. 24346722 10.1590/s0034-8910.2013047003807

[24] Ljungman S Granerus G The evaluation of kidney function in hypertensive patients. In [JH L, BM B, editors] Hypertension: pathophysiology, diagnosis, and management. 2nd ed., pp. 1987–2004. New York 1995.

[25] Mill JG Pinto K Griep RH Aferições e exames clínicos realizados nos participantes do ELSA-Brasil. Rev Saúde Pública 2013;47:54–62. 24346721 10.1590/s0034-8910.2013047003851

[26] Chor D de M Alves MG Questionnaire development in ELSA-Brasil: challenges of a multidimensional instrument. J Public Health 2013;47:27–36. 10.1590/s0034-8910.2013047003835 24346718

[27] Matsudo S Araújo T Matsudo V Questionário Internacional De Atividade Física (IPAQ): Estudo de Validade e Reprodutibilidade no Brasil. Rev Bras Ativ Fís Saúde 2001;6:5–18.

[28] Perez V Chang ET Sodium-to-Potassium ratio and blood pressure, hypertension, and related factors. Adv Nutr 2014;5:712–41. 25398734 10.3945/an.114.006783 PMC4224208

[29] Huggins CE O’Reilly S Brinkman M Relationship of urinary sodium and sodium-to-potassium ratio to blood pressure in older adults in Australia. Med J Aust 2011;195:128–32. 21806530 10.5694/j.1326-5377.2011.tb03239.x

[30] Polónia J Maldonado J Ramos R Estimation of salt intake by urinary sodium excretion in a Portuguese adult population and its relationship to arterial stiffness. Rev Port Cardiol 2006;25:801–17. 17100171

[31] Chien K Hsu H Chen P-C Urinary sodium and potassium excretion and risk of hypertension in Chinese: report from a community-based cohort study in Taiwan. J Hypertens 2008;26:1750–6. 18698208 10.1097/HJH.0b013e328306a0a7

[32] Frisoli TM Schmieder RE Grodzicki T Salt and hypertension: Is salt dietary reduction worth the effort? Am J Med 2012;125:433–9. 22482843 10.1016/j.amjmed.2011.10.023

[33] Silva ABT Capingana DP Magalhães P Predictors and reference values of pulse wave velocity in prepubertal angolan children. J Clin Hypertens 2016;18:725–32. 10.1111/jch.12739 PMC8031821 26663634

[34] Bernstein AM Willett WC Trends in 24-h urinary sodium excretion in the United States, 1957–2003: a systematic review. Am J Clin Nutr 2010;92:1172–80. 20826631 10.3945/ajcn.2010.29367 PMC2954449

[35] Aburto NJ Hanson S Gutierrez H Effect of increased potassium intake on cardiovascular risk factors and disease: systematic review and meta-analyses. BMJ 2013;346:f1378. 23558164 10.1136/bmj.f1378 PMC4816263

[36] Filippini T Violi F D’Amico R The effect of potassium supplementation on blood pressure in hypertensive subjects: A systematic review and meta-analysis. Int J Cardiol 2017;230:127–35. 28024910 10.1016/j.ijcard.2016.12.048

[37] D’Elia L Iannotta C Sabino P Potassium-rich diet and risk of stroke: updated meta-analysis. Nutr Metab Cardiovasc Dis 2014;24:585–7. 24780514 10.1016/j.numecd.2014.03.001

[38] Rodriguez-Rodriguez E Ortega RM Andres Carvajales P Relationship between 24 h urinary potassium and diet quality in the adult Spanish population. Public Health Nutr 2015;18:850–9. 25075534 10.1017/S1368980014001402 PMC10271480

[39] Wu L Sun D He Y Fruit and vegetables consumption and incident hypertension: dose-response meta-analysis of prospective cohort studies. J Hum Hypertens 2016;30:573–80. 27306085 10.1038/jhh.2016.44

[40] Song HJ Paek YJ Choi MK Gender differences in the relationship between risk of hypertension and fruit intake. Prev Med 2014;67:154–9. 25045835 10.1016/j.ypmed.2014.07.016

[41] Boeing H Bechthold A Bub A Critical review: vegetables and fruit in the prevention of chronic diseases. Eur J Nutr 2012;51:637–63. 22684631 10.1007/s00394-012-0380-y PMC3419346

[42] Miura K Greenland P Stamler J Relation of vegetable, fruit, and meat intake to 7-year blood pressure change in middle-aged men: the chicago western electric study. Am J Epidemiol 2004;159:572–80. 15003961 10.1093/aje/kwh085

[43] He FJ Nowson CA MacGregor GA Fruit and vegetable consumption and stroke: meta-analysis of cohort studies. Lancet 2006;367:320–6. 16443039 10.1016/S0140-6736(06)68069-0

[44] Dauchet L Amouyel P Hercberg S Fruit and vegetable consumption and risk of coronary heart disease: a meta-analysis of cohort studies. J Nutr 2006;136:2588–93. 16988131 10.1093/jn/136.10.2588

[45] Park J Kwock C Yang Y The effect of the sodium to potassium ratio on hypertension prevalence: a propensity score matching approach. Nutrients 2016;8:482. 10.3390/nu8080482 PMC4997395 27509520

[46] Rodrigues SL Baldo MP Machado RC High potassium intake blunts the effect of elevated sodium intake on blood pressure levels. J Am Soc Hypertens 2014;8:232–8. 24524886 10.1016/j.jash.2014.01.001

[47] Potischman N Biologic and methodologic issues for nutritional biomarkers. J Nutr 2003;133:875S–80S. 12612173 10.1093/jn/133.3.875S

[48] Cogswell ME Maalouf J Elliott P Use of urine biomarkers to assess sodium intake: challenges and opportunities. Annu Rev Nutr 2015;35:349–87. 25974702 10.1146/annurev-nutr-071714-034322 PMC5497310

[49] Pereira TSS Cade NV Mill JG Use of the method of triads in the validation of sodium and potassium intake in the brazilian longitudinal study of adult health (ELSA-Brasil). Plos One 2016;11:e0169085. 28030625 10.1371/journal.pone.0169085 PMC5193445

[50] Schmidt MI Griep RH Passos VM Strategies and development of quality assurance and control in the ELSA-Brasil. Rev Saúde Pública 2013;47:105–12. 24346727 10.1590/s0034-8910.2013047003889

